# Supplementary material for: Modeling glioblastoma heterogeneity as a dynamic network of cell states
Source: Mol Syst Biol. 2021 Sep 16;17(9):e10105. doi: 10.15252/msb.202010105 (PMC8444284; doi:10.15252/msb.202010105)
Supplement: Supplementary file 5 — Source Data for Figure 3 [file MSB-17-e10105-s001.zip › Figure3A_sourcedata/GSEA_3065/hallmarks_state5.GseaPreranked.1623416302988/HALLMARK_ANGIOGENESIS.html]

Details for gene set HALLMARK\_ANGIOGENESIS[GSEA]

|  || Dataset | state5 |
| Phenotype | NoPhenotypeAvailable |
| Upregulated in class | na\_pos |
| GeneSet | HALLMARK\_ANGIOGENESIS |
| Enrichment Score (ES) | 0.7401819 |
| Normalized Enrichment Score (NES) | 2.2043014 |
| Nominal p-value | 0.0 |
| FDR q-value | 0.0 |
| FWER p-Value | 0.0 |
Table: GSEA Results Summary

  

Fig 1: Enrichment plot: HALLMARK\_ANGIOGENESIS      
 Profile of the Running ES Score & Positions of GeneSet Members on the Rank Ordered List

  

| PROBE | GENE SYMBOL | GENE\_TITLE | RANK IN GENE LIST | RANK METRIC SCORE | RUNNING ES | CORE ENRICHMENT || 1 | APP |  |  | 38 | 0.712 | 0.1212 | Yes |
| 2 | LRPAP1 |  |  | 57 | 0.619 | 0.2282 | Yes |
| 3 | TIMP1 |  |  | 109 | 0.518 | 0.3138 | Yes |
| 4 | ITGAV |  |  | 118 | 0.508 | 0.4024 | Yes |
| 5 | VCAN |  |  | 191 | 0.444 | 0.4727 | Yes |
| 6 | POSTN |  |  | 316 | 0.375 | 0.5249 | Yes |
| 7 | COL5A2 |  |  | 407 | 0.337 | 0.5743 | Yes |
| 8 | STC1 |  |  | 530 | 0.303 | 0.6141 | Yes |
| 9 | FGFR1 |  |  | 545 | 0.299 | 0.6652 | Yes |
| 10 | LPL |  |  | 646 | 0.276 | 0.7027 | Yes |
| 11 | SPP1 |  |  | 724 | 0.256 | 0.7393 | Yes |
| 12 | FSTL1 |  |  | 1086 | 0.200 | 0.7343 | Yes |
| 13 | JAG1 |  |  | 1304 | 0.170 | 0.7402 | Yes |
| 14 | VEGFA |  |  | 1634 | 0.139 | 0.7279 | No |
| 15 | PDGFA |  |  | 2967 | 0.054 | 0.5888 | No |
| 16 | PTK2 |  |  | 2987 | 0.052 | 0.5959 | No |
| 17 | VAV2 |  |  | 3172 | 0.043 | 0.5829 | No |
| 18 | NRP1 |  |  | 3735 | 0.019 | 0.5235 | No |
| 19 | CCND2 |  |  | 4539 | -0.016 | 0.4367 | No |
| 20 | MSX1 |  |  | 6868 | -0.127 | 0.1994 | No |
| 21 | TNFRSF21 |  |  | 7627 | -0.208 | 0.1514 | No |
Table: GSEA details [plain text format]

  

Fig 2: HALLMARK\_ANGIOGENESIS: Random ES distribution      
 Gene set null distribution of ES for **HALLMARK\_ANGIOGENESIS**
